# Supplementary material for: Illuminating the function of the orphan transporter, SLC22A10, in humans and other primates
Source: Nat Commun. 2024 May 23;15:4380. doi: 10.1038/s41467-024-48569-7 (PMC11116522; doi:10.1038/s41467-024-48569-7)
Supplement: Supplementary file 1 — Supplementary Information [file 41467_2024_48569_MOESM1_ESM.pdf]

Illuminating the Function of the Orphan Transporter, SLC22A10, in Humans and Other Primates

Sook Wah Yee, Luis Ferrández-Peral, Pol Alentorn-Moron, Claudia Fontsere, Merve Ceylan, Megan L. Koleske, Niklas Handin, Virginia M. Artegoitia, Giovanni Lara, Huan-Chieh Chien, Xujia Zhou, Jacques Dainat, Arthur Zalevsky, Andrej Sali, Colin M. Brand, Finn D. Wolfreys, Jia Yang, Jason E. Gestwicki, John A. Capra, Per Artursson, John W. Newman, Tomas Marques-Bonet, Kathleen M. Giacomini

Supplementary Information

Supplementary Figure 1

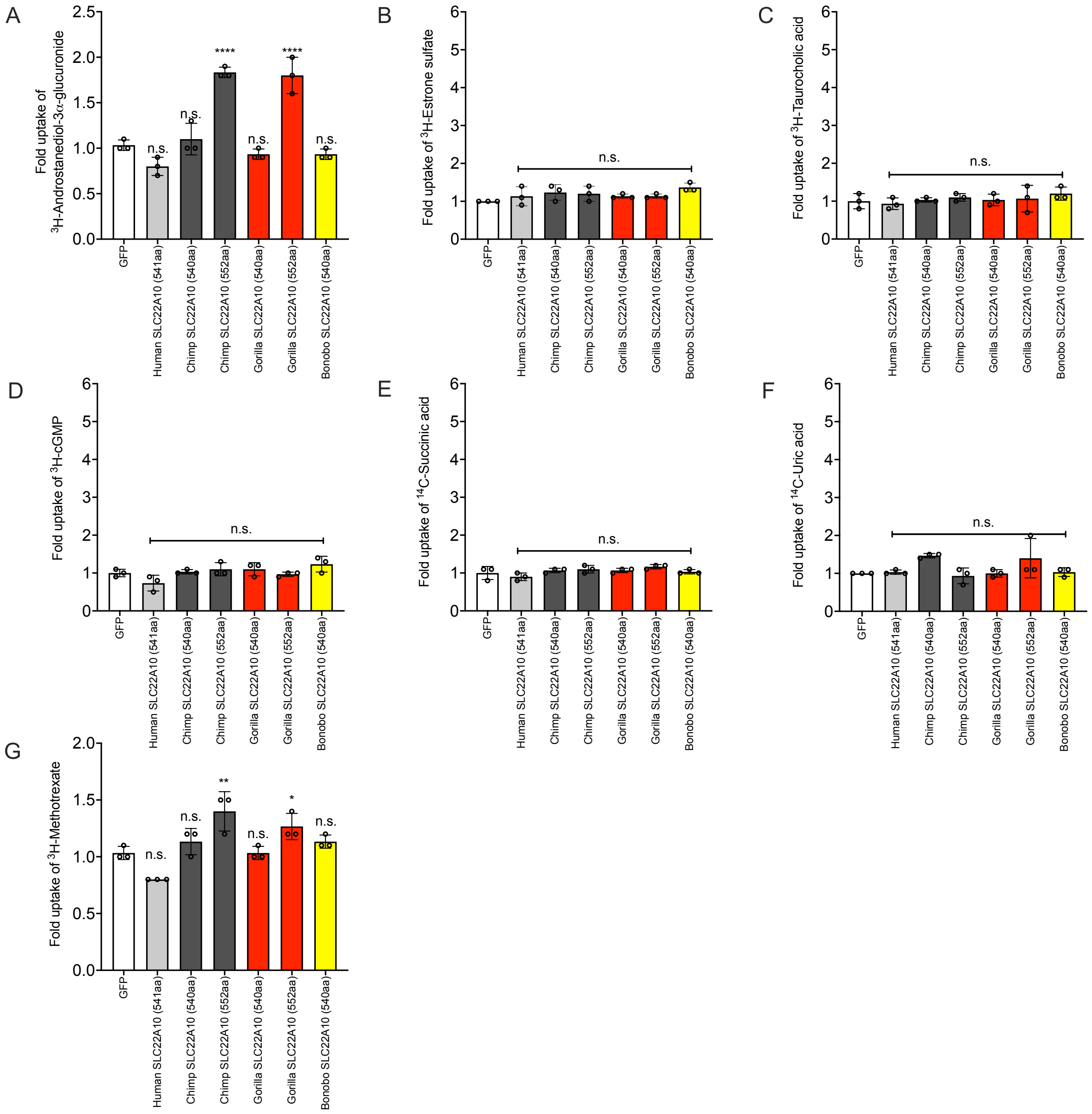

Supplementary Figure 1. Six anions were screened as substrates of SLC22A10. (A) [^{3}H]-androstanediol-3α-glucuronide; (B) [^{3}H]-estrone sulfate; (C) [^{3}H]-taurocholic acid; (D) [^{3}H]-cGMP; (E) [^{14}C]-succinic acid, (F) [^{14}C]-uric acid, (G) [^{3}H]-methotrexate. HEK293 cells transiently transfected with GFP vector was used as negative control. All expression vectors used have GFP-tagged in the N-terminal. Statistical significance was determined using a one-way analysis of variance (ANOVA) with Dunnett's multiple comparison test to compare the mean of each orthologs with the mean of the negative control (GFP). Data are from one representative experiment in triplicate wells (mean ± s.d.). n.s.: not significant, ****$p<0.0001$, ***$p<0.0005$, **$p<0.01$, *$p<0.05$. The scatter plot with bars displayed the mean +/- standard deviation of three technical replicates (n=1 shown as a representative experiment). Source data

are provided as a Source Data file. Similar results were obtained in two independent experiments.

Supplementary Figure 2

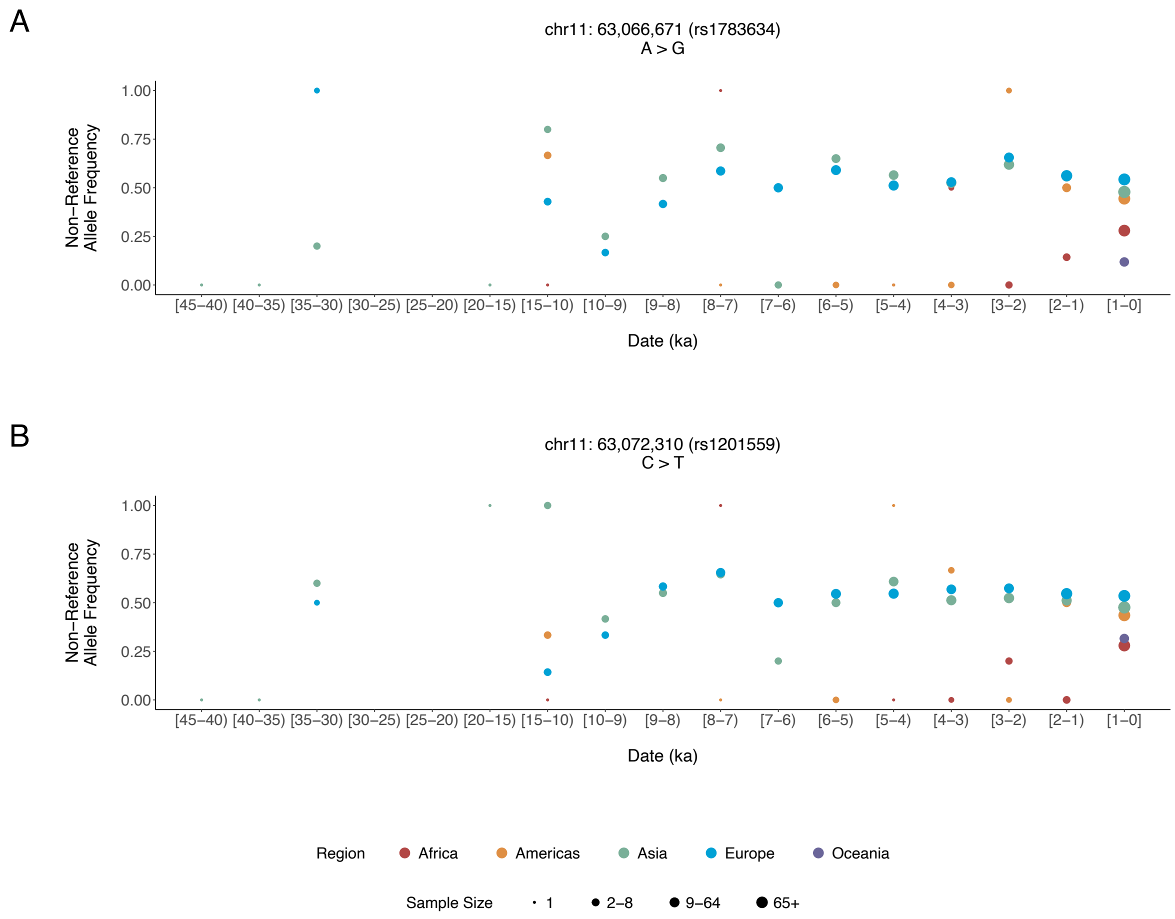

Supplementary Figure 2. Allele frequencies of two single nucleotide polymorphisms (SNPs), rs1783634 and rs1201559, were analyzed in diverse human populations. A. The allele frequency of rs1783634, stratified by time period and geographic location of the sample. This SNP is highly correlated (D' = 1, r^2 = 0.9839) with SLC22A10-Trp96Ter (rs1790218). Allele frequencies were calculated using ancient and modern data from the Allen Ancient DNA Resources (AADR) (Mallick et al. 2023). B. The allele frequency of rs1201559, another SNP highly correlated with rs1790218 (D' = 0.9975, r2 =0.9775), stratified by time period and geographic location of the sample. Data are from the same reference and visualized as in A. The allele frequencies for these two SNPs were obtained from The Simons Genome Diversity Project (https://www.simonsfoundation.org/simons-genome-diversity-project/), 1000 Genomes Project, and the Allen Ancient DNA Resources (AADR) (https://reich.hms.harvard.edu/allen-ancient-dna-resource-aadr-downloadable-genotypes-present-day-and-ancient-dna-data).

Supplementary Figure 3

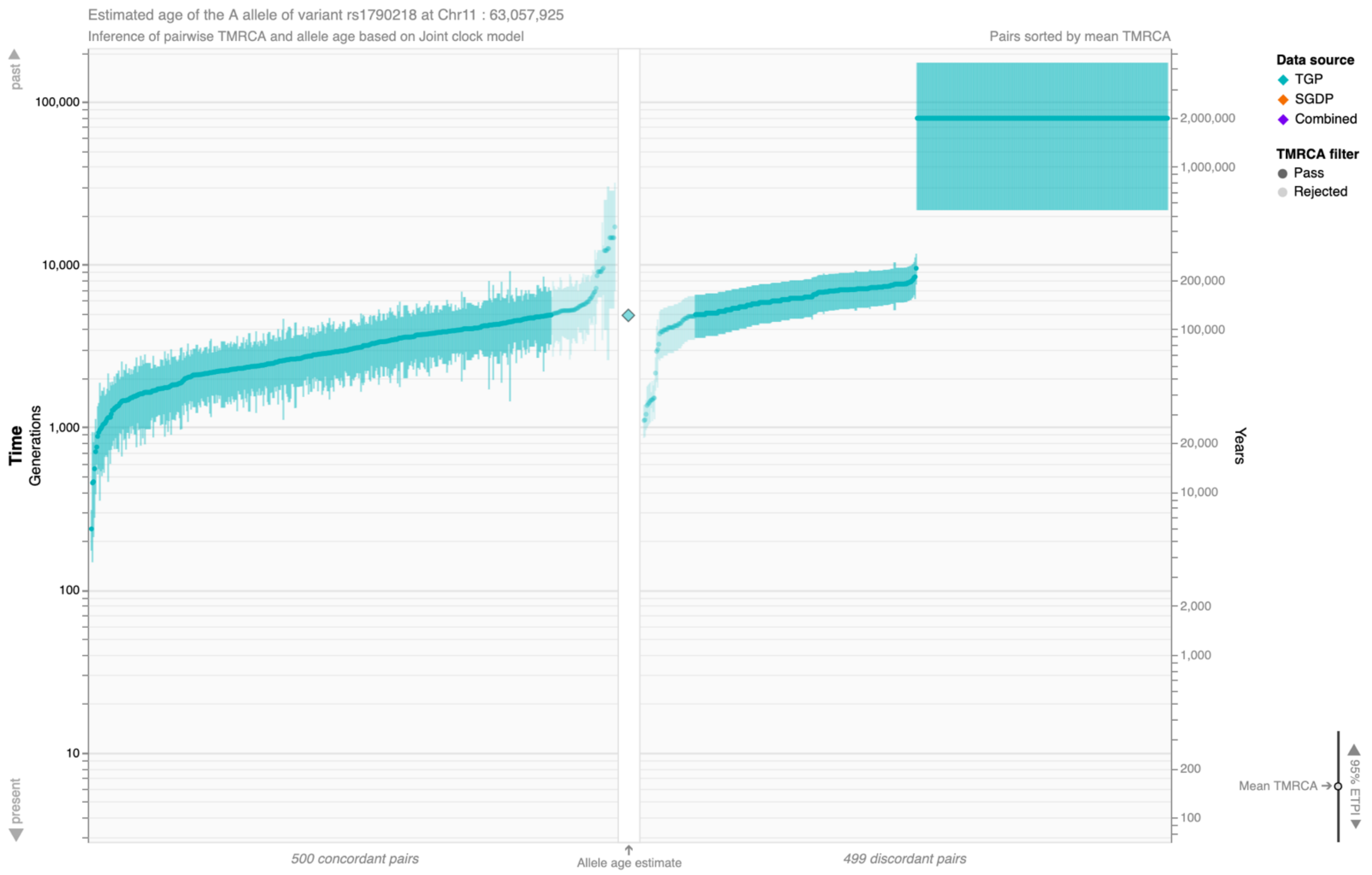

Supplementary Figure 3. Allele age estimate for rs1790218 (G > A) from the Human Genome Dating portal using the joint clock model. The A allele is estimated to emerge 4,873 generations or 121,825 years ago (quality score = 0.878). ETPI = equal-tailed probability interval (95% credible interval), SGDP = Simons Genome Diversity Project, TGP = 1000 Genomes Project, TMRCA = time to most recent common ancestor.

Supplementary Figure 4

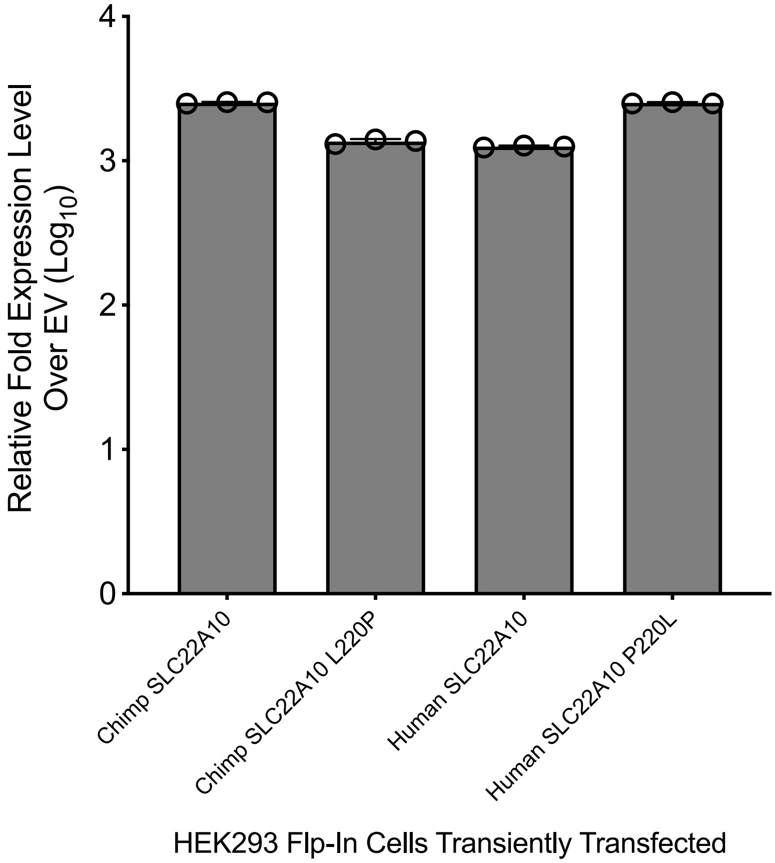

Supplementary Figure 4. Transcript levels of SLC22A10 in HEK293 cells that were transiently transfected with human and chimpanzee SLC22A10, as well as their respective mutations. The scatter plot with bars displayed the mean +/- standard deviation of three technical replicates (n=1 shown as a representative experiment). Source data are provided as a Source Data file.

Supplementary Figure 5

A

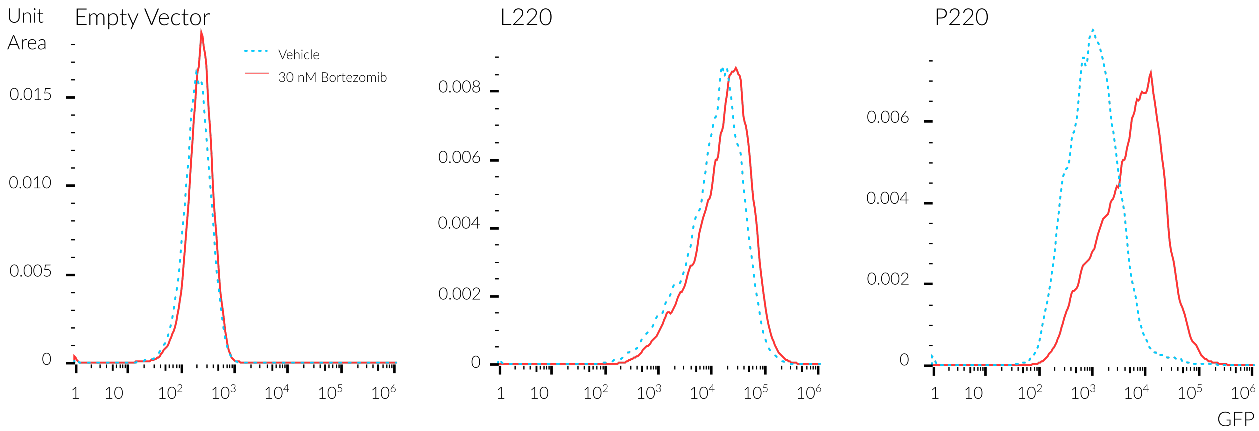

B

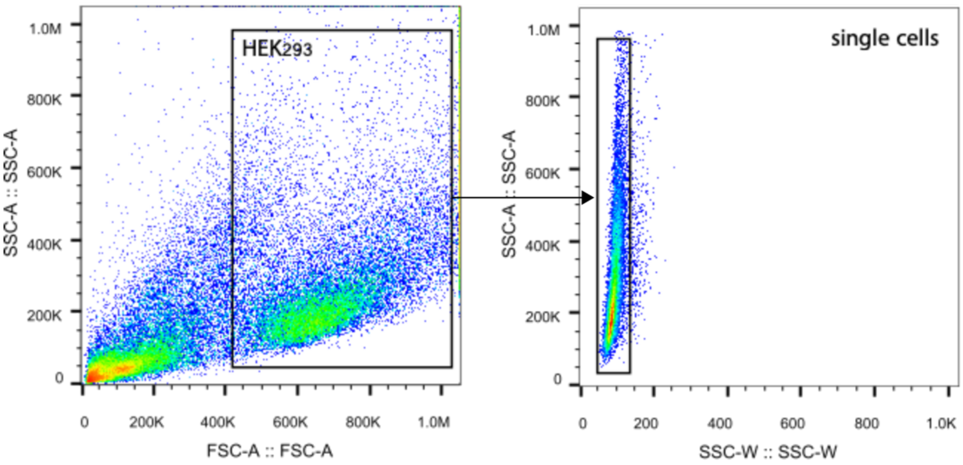

Supplementary Figure 5. A. Histograms of GFP fluorescence in HEK293 Flp-In cells stably transfected with empty vector control, SLC22A10 L220 mutant, and SLC22A10 reference (P220) GFP fusions treated with DMSO vehicle and 30 nM bortezomib. Increased GFP fluorescence upon treatment of SLC22A10-P220 indicates accumulation of the SLC22A10 that would otherwise have been rapidly degraded -- likely a consequence of its inherent instability. The blue histogram represents the distribution after treatment with the DMSO vehicle, and the red histogram represents the distribution after treatment with 30 nM bortezomib for 16 hours (n = 1 shown as a representative experiment from one replicate). Similar results were obtained in two independent experiments. B. Representative gating strategy for cells shown in above histogram.

Supplementary Figure 6

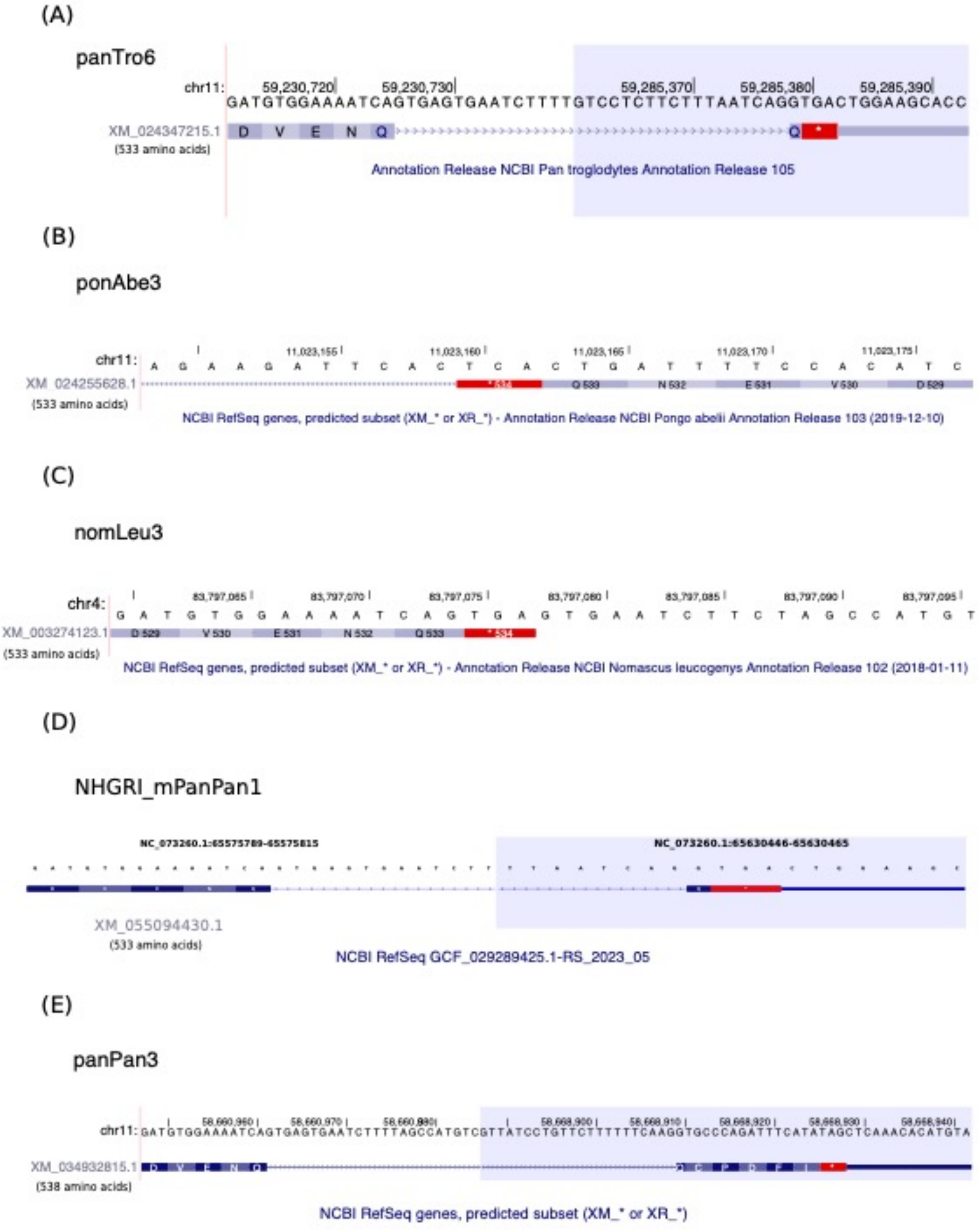

Supplementary Figure 6. SLC22A10 isoforms encoding 533 amino acids in chimpanzee, orangutan, gibbon and bonobo. In addition to the 552 amino acids SLC22A10, NCBI annotations show that chimpanzee (A), orangutan (B), and gibbon (C) and bonobo (D) are predicted to have isoforms with 533 amino acids. It is worth noting that at the beginning of this project, NCBI had initially predicted the bonobo SLC22A10 isoform to have 538 amino acids (panPan3 assembly) (E). These shorter protein isoforms are derived from alternative acceptor sites (chimpanzee/bonobo) and exon extensions (orangutan/gibbon). Blue shadows indicate a split view of the intron sequence between two exons.

Supplementary Figure 7

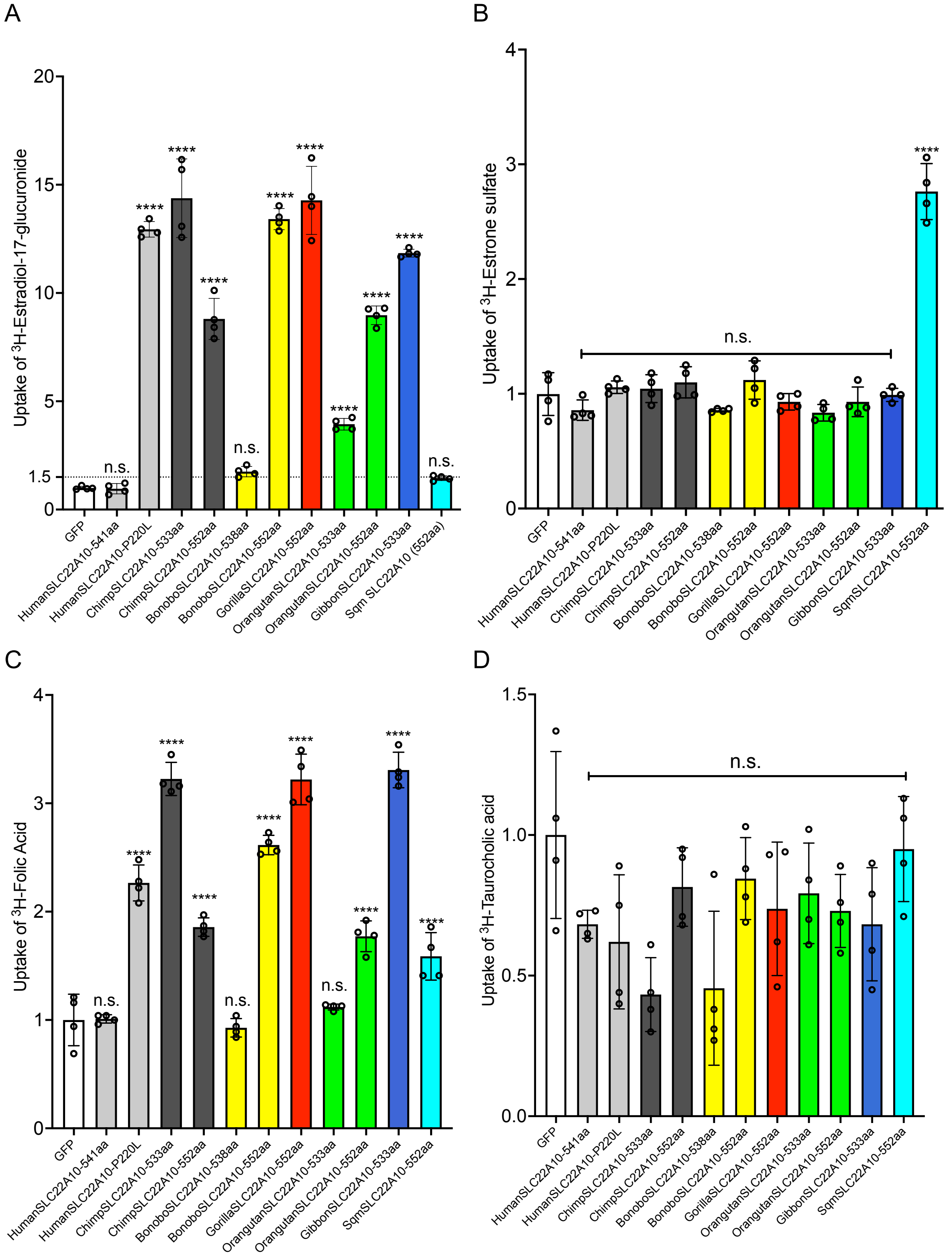

Supplementary Figure 7. Four anions were screened as substrates of SLC22A10 of different species and isoforms. Human SLC22A10 and human SLC22A10-P220L are also included in the assay. A. [^{3}H]-estradiol-17β-glucuronide; B. [^{3}H]-estrone sulfate; C. [^{3}H]-folic acid; and D. [^{3}H]-taurocholic acid. HEK293 cells transiently transfected with GFP

vector was used as control. The plot displays the mean +/- standard deviation of four technical replicates (n = 1 shown as a representative experiment). The expression vectors used in this experiment are not GFP-tagged. Similar results were obtained in two independent experiments. Source data are provided as a Source Data file. n.s. Not significance compared to HEK293 cells transfected with GFP only. Statistical significance was determined using a one-way analysis of variance (ANOVA) with Dunnett's multiple comparison test to compare the mean of each orthologs with the mean of the negative control (GFP). n.s.: not significant, ****$p<0.0001$, ***$p<0.0005$, **$p<0.01$, *$p<0.05$. SqmSLC22A10-552aa: This is the cDNA sequence of SLC22A10 from the Squirrel Monkey, encoding 552 amino acids (ENSSBOT00000021267.1, Uniprot: A0A2K6SBP5).

Supplementary Figure 8

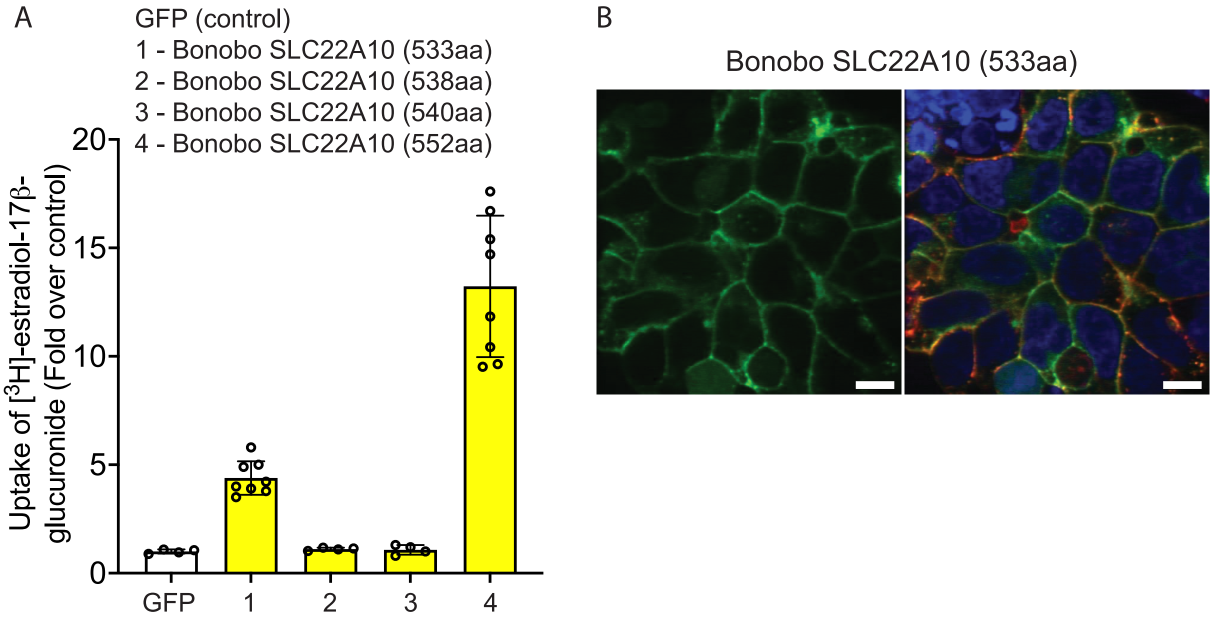

Supplementary Figure 8. Uptake and membrane localization of Bonobo SLC22A10 consists of 533 amino acids. **A.** Bonobo SLC22A10 consists of 533 amino acids exhibited a significant uptake of [^{3}H]-estradiol-17β-glucuronide and demonstrated plasma localization. The expression vectors used in this experiment are GFP-tagged. HEK293 Flp-In cells transiently transfected with GFP vector was used as control. The statistical significance was determined using a one-way analysis of variance (ANOVA) with Dunnett's multiple comparison test to compare the mean of each isoform with that of the negative control (GFP). n.s. Not significance compared to HEK293 Flp-In cells transfected with GFP only. ****p<0.0001, *p<0.05, n.s. not significance. Data are from one representative experiment. Graphs depict mean +/- standard deviation and points represent technical replicates. **B.** Localization of bonobo SLC22A10 conjugated to green fluorescent protein (GFP) was examined in HEK293 cells using high-content imaging and cellular staining with the plasma membrane marker wheat germ agglutinin (WGA). Blue: DNA stain Hoechst marks the cell nucleus; Red: Plasma membrane marker WGA; Green: SLC22A10. Yellow: Merge. The results showed colocalization of GFP-tagged SLC22A10 with WGA. The image shows a representative image from two technical replicate experiments. Scale bar: 10 μM.

Supplementary Figure 9

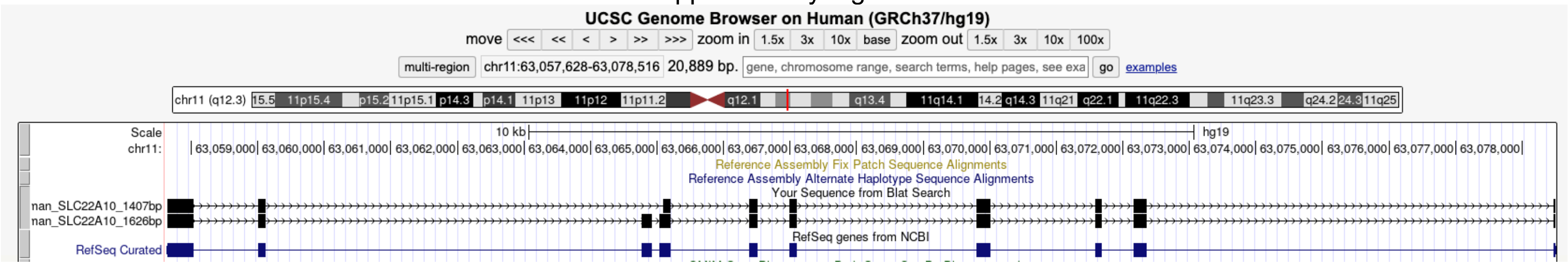

>human SLC22A10 open reading frame (three out of 27 colonies have the full SLC22A10 ORF)
ATGGCCTTTGAGGAGCTCTTGAGTCAAGTTGGAGGCCTTGGGAGATTTCAGATGCTTCATCTGGTTTTTATTCTTCCCTCTCTCAT
GTTATTAATCCCTCATATACTGCTAGAGAACTTTGCTGCAGCCATTCCTGGTCATCGTTGCTGGGTCCACATGCTGGACAATAATA
CTGGATCTGGTAATGAAACTGGAATCCTCAGTGAAGATGCCCTCTTGAGAATCTCTATCCCACTAGACTCAAATCTGAGGCCAGA
GAAGTGTCGTCGCTTTGTCCATCCCCAGTGGCAGCTTCTTCACCTGAATGGGACTATCCACAGCACAAGTGAGGCAGACACAGA
ACCCTGTGTGGATGGCTGGGTATATGATCAAAGCTACTTCCCTTCGACCATTGTGACTAAGTGGGACCTGGTATGTGATTATCAG
TCACTGAAATCAGTGGTTCAATTCCTACTTCTGACTGGAATGCTGGTGGGAGGCATCATAGGTGGCCATGTCTCAGACAGGTTTG
GGCGAAGATTTATTCTCAGATGGTGTTTGCTCCAGCTTGCCATTACTGACACCTGCGCTGCCTTCGCTCCCACCTTCCCTGTTTA
CTGTGTACTACGCTTCTTGGCAGGTTTTTCTTCCATGATCATTATATCAAATAATTCTTTGCCCATTACTGAGTGGATAAGGCCCA
ACTCTAAAGCCCTGGTAGTAATATTGTCATCTGGTGCCCTTAGTATTGGACAGATAATCCTGGGAGGCTTGGCTTATGTCTTCCG
AGACTGGCAAACCCTGCACGTGGTGGCGTCTGTACCTTTCTTTGTCTTCTTTCTTCTTTCAAGGTGGCTGGTGGAATCTGCTCGG
TGGTTGATAATCACCAATAAACTAGATGAGGGCTTAAAGGCACTTAGAAAAGTTGCACGCACAAATGGAATAAAGAATGCTGAAG
AAACCCTGAACATAGAGGTTGTAAGATCCACCATGCAGGAGGAGCTGGATGCAGCACAGACCAAAACTACTGTGTGTGACTTGT
TCCGCAACCCCAGTATGCGTAAAAGGATCTGTATCCTGGTATTTTTGAGATTTGCAAACACAATACCTTTTTATGGTACCATGGTC
AATCTTCAGCATGTGGGGAGCAACATTTTCCTGTTGCAGGTACTTTATGGAGCTGTCGCTCTCATAGTTCGATGTCTTGCTCTTTT
GACACTAAATCATATGGGCCGTCGAATAAGCCAGATATTGTTCATGTTCCTGGTGGGCCTTTCCATTTTGGCCAACACGTTTGTG
CCCAAAGAAATGCAGACCCTGCGTGTGGCTTTGGCATGTCTGGGAATCGGCTGTTCTGCTGCTACTTTTTCCAGTGTTGCTGTTC
ACTTCATTGAACTCATCCCCACTGTTCTCAGGGCAAGAGCTTCAGGAATAGATTTAACGGCTAGTAGGATTGGAGCAGCACTGG
CTCCCCTCTTGATGACCTTAACGGTATTTTTTACCACTTTGCCATGGATCATTTATGGAATCTTCCCCATCATTGGTGGCCTTATT
GTCTTCCTCCTACCAGAAACCAAGAATCTGCCTTTGCCTGACACCATCAAGGATGTGGAAAATCAAAAAAAAAATCTCAAGGAAA
AGGCATAA

>human SLC22A10 open reading frame (23 out of 27 colonies have the 219-bp deletion (in red) of the SLC22A10 ORF)
ATGGCCTTTGAGGAGCTCTTGAGTCAAGTTGGAGGCCTTGGGAGATTTCAGATGCTTCATCTGGTTTTTATTCTTCCCTCTCTCAT
GTTATTAATCCCTCATATACTGCTAGAGAACTTTGCTGCAGCCATTCCTGGTCATCGTTGCTGGGTCCACATGCTGGACAATAATA
CTGGATCTGGTAATGAAACTGGAATCCTCAGTGAAGATGCCCTCTTGAGAATCTCTATCCCACTAGACTCAAATCTGAGGCCAGA

GAAGTGTCGTCGCTTTGTCCATCCCCAGTGGCAGCTTCTTCACCTGAATGGGACTATCCACAGCACAAGTGAGGCAGACACAGA
ACCCTGTGTGGATGGCTGGGTATATGATCAAAGCTACTTCCCTTCGACCATTGTGACTAAGTGGGACCTGGTATGTGATTATCAG
TCACTGAAATCAGTGGTTCAATTCCTACTTCTGACTGGAATGCTGGTGGGAGGCATCATAGGTGGCCATGTCTCAGACAGGTTTG
GGCGAAGATTTATTCTCAGATGGTGTTTGCTCCAGCTTGCCATTACTGACACCTGCGCTGCCTTCGCTCCCACCTTCCCTGTTTA
CTGTGTACTACGCTTCTTGGCAGGTTTTTCTTCCATGATCATTATATCAAATAATTCTTTGCCCATTACTGAGTGGATAAGGCCCA
ACTCTAAAGCCCTGGTAGTAATATTGTCATCTGGTGCCCTTAGTATTGGACAGATAATCCTGGGAGGCTTGGCTTATGTCTTCCG
AGACTGGCAAACCCTGCACGTGGTGGCGTCTGTACCTTTCTTTGTCTTCTTTCTTCTTTCAAGGTGGCTGGTGGAATCTGCTCGG
TGGTTGATAATCACCAATAAACTAGATGAGGGCTTAAAGGCACTTAGAAAAGTTGCACGCACAAATGGAATAAAGAATGCTGAAG
AAACCCTGAACATAGAGGTTGTAAGATCCACCATGCAGGAGGAGCTGGATGCAGCACAGACCAAAACTACTGTGTGTGACTTGT
TCCGCAACCCCAGTATGCGTAAAAGGATCTGTATCCTGGTATTTTTGAGATTTGCAAACACAATACCTTTTTATGGTACCATGGTC
AATCTTCAGCATGTGGGGAGCAACATTTTCCTGTTGCAGGTACTTTATGGAGCTGTCGCTCTCATAGTTCGATGTCTTGCTCTTTT
GACACTAAATCATATGGGCCGTCGAATAAGCCAGATATTGTTCATGTTCCTGGTGGGCCTTTCCATTTTGGCCAACACGTTTGTG
CCCAAAGAAATGCAGACCCTGCGTGTGGCTTTGGCATGTCTGGGAATCGGCTGTTCTGCTGCTACTTTTTCCAGTGTTGCTGTTC
ACTTCATTGAACTCATCCCCACTGTTCTCAGGGCAAGAGCTTCAGGAATAGATTTAACGGCTAGTAGGATTGGAGCAGCACTGG
CTCCCCTCTTGATGACCTTAACGGTATTTTTTACCACTTTGCCATGGATCATTTATGGAATCTTCCCCATCATTGGTGGCCTTATT
GTCTTCCTCCTACCAGAAACCAAGAATCTGCCTTTGCCTGACACCATCAAGGATGTGGAAAATCAAAAAAAAAATCTCAAGGAAA
AGGCATAA

Supplementary Figure 9. Human SLC22A10 open reading frame (ORF). The first fasta sequence above is human SLC22A10 ORF with 1626 bp (NM_001039752). The second fasta sequence has a 219-bp deletion (in red), result in 1407 bp. The two fasta sequence were pasted in UCSC genome browser (hg19) to Blat search. The human SLC22A10 ORF with 1407bp skip the entire exon 3 and early part of exon 4. The sequences of the two human SLC22A10 sequence are available in Data Source file.

Supplementary Figure 10

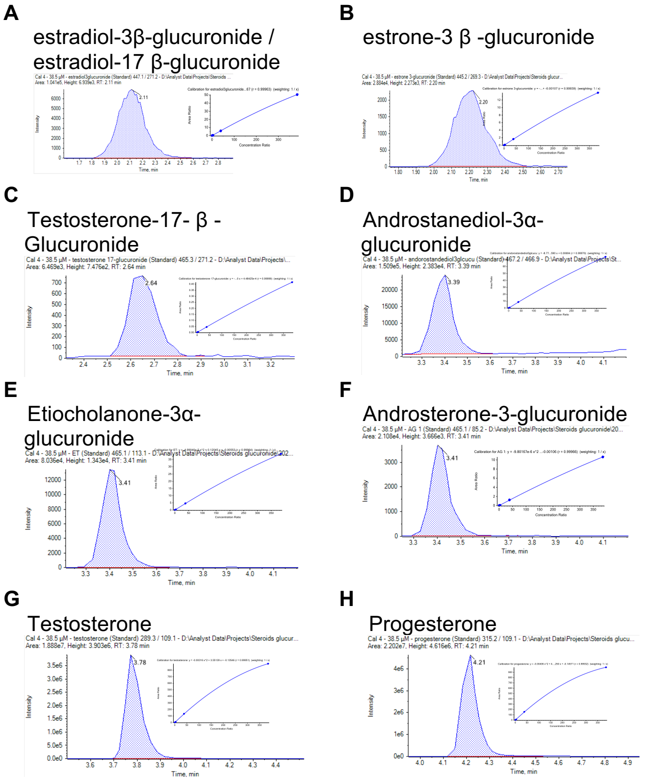

Supplementary Figure 10. Representative chromatograms of steroid metabolites and calibration curves. Mass transitions used for quantification are as follows: **A**) 447.1 >271.2 m/z; **B**) 445.2 >269.3 m/z; **C**) 465.3 > 271.2 m/z; **D**) 467.2 > 466.9 m/z; **E**) 467.1 > 113.1 m/z; **F**) 465.1 >85.2 m/z; **G**) 289.3 > 109.1 m/z; **H**) 315.2 > 109.1 m/z. Five-point calibration curves from 0.0385 – 385 µM are shown as insets on each chromatogram. For each metabolite, quadratic curve fits with 1/x weighting yielded coefficients of variation ≥ 0.9995. For positional estradiol-glucuronides (Panel **A**) and the etiocholanone-3α- and androsterone-3-glucuronides (Panel **E** & **F**), the isomers were not chromatographically resolved and the calibration curve represents the sum of the two compounds. To view enlarged images **A** to **H**, please refer to the chromatograms of steroids and steroid conjugates in the Data Source file.

Supplementary Table 1. Haplotype frequencies for rs1790218 and rs768117722 among genotypes from all Thousand Genomes populations calculated using the LDhap tool from LDlink.

GrCh37/hg19 Position	RS Number	Allele Frequencies	Haplotypes		
chr11: 63,057,925	rs1790218	G=0.567, A=0.433	G	A	G
chr11: 63,078,479	rs768117722	A=0.984, -=0.016	A	A	-
		Haplotype Count	2,756	2,171	81
		Haplotype Frequency	0.5503	0.4335	0.0162

Supplementary Table 2. List of steroid and steroid glucuronide tested.

**Compound**	**Vendor**	**Catalog number**
d3-estradiol-3α-glucuronide	Toronto Research Chemical	E888017
estradiol-3β-glucuronide	Sigma-Aldrich	E2127-5MG
estradiol-17β-glucuronide	Sigma-Aldrich	E1127-10mg
estrone-3β-glucuronide	Toronto Research Chemical	E889055
testosterone-17β-glucuronide	Steraloids	A6980-000
androstanediol-3α-glucuronide	Steraloids	A1206-800
etiocholanolone-3α-glucuronide	Steraloids	A3625-000
androsterone-3-glucuronide	Toronto Research Chemical	A637605
testosterone	Sigma-Aldrich	T1500
progesterone	Sigma-Aldrich	P0130

Supplementary Table 3. Steroid and steroid glucuronide mass spectrometer acquisition parameters.

compound	tR (min)	ESI Mode	Q1 (Da)	Q3 (Da)	DP	CE
d3-estradiol-3α-glucuronide	1.5	(-)	450.1	274.2	-25	-25
estradiol-3β-glucuronide	1.90	(-)	447.2	271.2	-25	-25
estradiol-17β-glucuronide	1.94	(-)	447.2	271.2	-25	-40
estrone-3β-glucuronide	2.00	(-)	445.2	269.2	-25	-20
testosterone-17β-glucuronide	2.58	(+)	465.3	271.2	130	10
androstanediol-3α-glucuronide	3.44	(-)	467.2	467.2	-25	-6
etiocholanolone-3α-glucuronide	3.45	(-)	465.3	113.1	-25	-37
androsterone-3-glucuronide	3.46	(-)	465.3	85.2	-25	-40
testosterone	3.76	(+)	289.3	109.1	50	25
1-cyclohexyl-3-uriedo-decanoic acid	4.00	(+)	341.2	216.2	135	28
1-cyclohexyl-3-uriedo-decanoic acid	4.00	(-)	339.2	214.2	-140	-38
progesterone	4.20	(+)	315.2	109.1	40	28

The API 4500 source was operated at 650ºC, with spray voltage of 4500 V, curtain gas at 34 L/min, Gas 1at 50 L/min, Gas 2 at 20 L/min, and a collision gas setting of medium using nitrogen.
