# Supplementary material for: Illuminating the function of the orphan transporter, SLC22A10, in humans and other primates
Source: Nat Commun. 2024 May 23;15:4380. doi: 10.1038/s41467-024-48569-7 (PMC11116522; doi:10.1038/s41467-024-48569-7)
Supplement: Supplementary file 3 — Reporting Summary [file 41467_2024_48569_MOESM3_ESM.pdf]

nature portfolio

Corresponding author(s): Kathleen M. Giacomini

Last updated by author(s): Apr 10, 2024

Reporting Summary

Nature Portfolio wishes to improve the reproducibility of the work that we publish. This form provides structure for consistency and transparency in reporting. For further information on Nature Portfolio policies, see our Editorial Policies and the Editorial Policy Checklist.

Statistics

For all statistical analyses, confirm that the following items are present in the figure legend, table legend, main text, or Methods section.

n/a	Confirmed	
☐	☒	The exact sample size (*n*) for each experimental group/condition, given as a discrete number and unit of measurement
☐	☒	A statement on whether measurements were taken from distinct samples or whether the same sample was measured repeatedly
☐	☒	The statistical test(s) used AND whether they are one- or two-sided<br>*Only common tests should be described solely by name; describe more complex techniques in the Methods section.*
☒	☐	A description of all covariates tested
☒	☐	A description of any assumptions or corrections, such as tests of normality and adjustment for multiple comparisons
☐	☒	A full description of the statistical parameters including central tendency (e.g. means) or other basic estimates (e.g. regression coefficient) AND variation (e.g. standard deviation) or associated estimates of uncertainty (e.g. confidence intervals)
☐	☒	For null hypothesis testing, the test statistic (e.g. *F*, *t*, *r*) with confidence intervals, effect sizes, degrees of freedom and *P* value noted<br>*Give P values as exact values whenever suitable.*
☒	☐	For Bayesian analysis, information on the choice of priors and Markov chain Monte Carlo settings
☒	☐	For hierarchical and complex designs, identification of the appropriate level for tests and full reporting of outcomes
☒	☐	Estimates of effect sizes (e.g. Cohen's *d*, Pearson's *r*), indicating how they were calculated

Our web collection on statistics for biologists contains articles on many of the points above.

Software and code

Policy information about availability of computer code

Data collection	Graph Pad Prism 9 (for plotting and statistical t-test or ANOVA analysis). On-line EMBL-EBI Clustal Omega was used to perform multiple sequence alignment. ITOL was used to generate the phylogeny tree,h ttps://itol.embl.de/. FlowJo software was used to generate the figure for flow cytometry analysis.
Data analysis	Nonhuman primates' sequencing data was processed as in de Manuel et al (2016) and also that reads were manually inspected to confirm the variant calling. Also,R NA-seq reads from Ruiz-Orera et al (2015) were mapped to hg38 using STAR mapper,and splice junction structures from human and chimpanzee liver samples were manually checked directly from the reads.

For manuscripts utilizing custom algorithms or software that are central to the research but not yet described in published literature, software must be made available to editors and reviewers. We strongly encourage code deposition in a community repository (e.g. GitHub). See the Nature Portfolio guidelines for submitting code & software for further information.

Data

Policy information about availability of data

All manuscripts must include a data availability statement. This statement should provide the following information, where applicable:

- Accession codes, unique identifiers, or web links for publicly available datasets
- A description of any restrictions on data availability
- For clinical datasets or third party data, please ensure that the statement adheres to our policy

When secondary data or publicly available datasets were used to support our research findings, we will include hyperlinks in the Data Availability section.
The global proteomics data for HEK293 Flp-In cell lines, stably transfected with human and chimpanzee SLC22A10 in both reference and mutant forms, are available at PRIDE (Proteomics IDEntifications Database), https://www.ebi.ac.uk/pride/. The project's accession number is PXD047102, [https://www.ebi.ac.uk/pride/archive/projects/PXD04710].. Archaic hominin genotypes for three Neanderthals and a Denisovan were retrieved from [http://ftp.eva.mpg.de/neandertal/Vindija/VCF/] and [http://ftp.eva.mpg.de/neandertal/Chagyrskaya/VCF/]. Ancient human genotypes from the Allen Ancient DNA Resource (AADR) were retrieved from this site [https://reichdata.hms.harvard.edu/pub/datasets/amh_repo/curated_releases/V54/V54.1.p1/SHARE/public.dir/]. The sequencing data of SLC22A10 in greater apes are obtained from these accession code:
PRJEB15086 [https://www.ebi.ac.uk/ena/browser/view/PRJEB15086], PRJEB19688 [https://www.ebi.ac.uk/ena/browser/view/PRJEB19688], PRJNA189439 [https://www.ebi.ac.uk/ena/browser/view/PRJNA189439].
The archived version of the code used to determine mutation effect predictions for the P220L variant of human SLC22A10 transporter has been deposited in Zenodo [https://doi.org/10.5281/zenodo.8411757]. The archived version of the code used to analyze ancient human genomes and visualize allele frequencies has been deposited in Zenodo [https://doi.org/10.5281/zenodo.10823210]. A non-archived version is available on GitHub [https://github.com/brandcm/SLC22A10_Variant_Evolutionary_History].

Research involving human participants, their data, or biological material

Policy information about studies with human participants or human data. See also policy information about sex, gender (identity/presentation), and sexual orientation and race, ethnicity and racism.

Reporting on sex and gender	Not applicable
Reporting on race, ethnicity, or other socially relevant groupings	Not applicable
Population characteristics	Not applicable
Recruitment	Not applicable
Ethics oversight	Not applicable

Note that full information on the approval of the study protocol must also be provided in the manuscript.

Field-specific reporting

Please select the one below that is the best fit for your research. If you are not sure, read the appropriate sections before making your selection.

☒ Life sciences ☐ Behavioural & social sciences ☐ Ecological, evolutionary & environmental sciences

For a reference copy of the document with all sections, see nature.com/documents/nr-reporting-summary-flat.pdf

Life sciences study design

All studies must disclose on these points even when the disclosure is negative.

Sample size	No sample size calculation was performed. Generally, when we conduct transporter assays, we replicate the study in three or four wells, and then repeat the same experiment on different days. This is a standard practice in membrane transporter field when performing transporter uptake and kinetic studies.
Data exclusions	When conducting transporter uptake studies to ascertain if a compound is a substrate of SLC22Al0, we include positive controls, for example, using a different cell line for which a substrate has been previously reported by our group or others. Another positive control could be employing a substrate that is known to have been reported as a substrate for specific cell lines. On the day of the experiment, if the positive control does not yield significant results, we will disregard the entire data set and repeat the experiment.
Replication	The transporter uptake studies were conducted multiple times on separate days to demonstrate that a substrate is significantly accumulated in HEK293-Flp-In cells transfected with SLC22A10 (from different species) compared to those transfected with an empty vector. Two or three independent experiments were conducted within 1 month. All attempts at replication were successful.
Randomization	The samples were not allocated into experimental groups. Randomization is not relevant to this study as the objective is to assess if a compound is a substrate of a specific transporter, rather than comparing outcomes between different groups or treatments. Randomization is

	typically used to minimize bias and confounding variables when there are multiple groups or treatments. However, in this study, the focus is on a singular, specific interaction - the uptake of a compound by a transporter. Hence, the experimental design involves only the testing of the compound's uptake by the transporter in question, making randomization unnecessary and irrelevant to the study's objective.
Blinding	This study were not blinded to group allocation during data collection and/or analysis. Blinding was not relevant to this study, however, we have asked random lab members to perform the study to determine reproducibility by other lab members.

Reporting for specific materials, systems and methods

We require information from authors about some types of materials, experimental systems and methods used in many studies. Here, indicate whether each material, system or method listed is relevant to your study. If you are not sure if a list item applies to your research, read the appropriate section before selecting a response.

Materials & experimental systems

n/a	Involved in the study
☒	☐ Antibodies
☐	☒ Eukaryotic cell lines
☒	☐ Palaeontology and archaeology
☒	☐ Animals and other organisms
☒	☐ Clinical data
☒	☐ Dual use research of concern
☒	☐ Plants

Methods

n/a	Involved in the study
☒	☐ ChIP-seq
☐	☒ Flow cytometry
☒	☐ MRI-based neuroimaging

Eukaryotic cell lines

Policy information about cell lines and Sex and Gender in Research

Cell line source(s)	HEK293 Flp-In cells [https://www.thermofisher.com/order/catalog/product/R7S007]. This cell line were originally sold by Invitrogen and that was when it was purchased and subsequently have been used and published in many publications from my research group.
Authentication	None of the cell lines used have been authenticated.
Mycoplasma contamination	We utilize this kit to verify that the HEK293 cells, which stably express our transporters of interest, are free of mycoplasma contamination. The kit has the catalog number CUL00IB and is named 'MycoProbe Mycoplasma Detection Kit.' It is available in a 96-well plate size and can be purchased from Fisher Scientific. All cell lines used in the experiemnts were tested negative for mycoplasma contamination.
Commonly misidentified lines (See ICLAC register)	No commonly misidentified lines were used in this study.

Flow Cytometry

Plots

Confirm that:

☒ The axis labels state the marker and fluorochrome used (e.g. CD4-FITC).

☒ The axis scales are clearly visible. Include numbers along axes only for bottom left plot of group (a 'group' is an analysis of identical markers).

☒ All plots are contour plots with outliers or pseudocolor plots.

☐ A numerical value for number of cells or percentage (with statistics) is provided.

Methodology

Sample preparation	Cultured cell lines (HEK293 Flp-In cells transfected with different gene and tagged with GFP); Samples were treated with or without bortezomib for 16 hours and then collected in PBS-EDTA.
Instrument	Attune NxT Acoustic Focusing Cytometer (Life Technologies)
Software	FlowJo v10 Software
Cell population abundance	Cells were grown in a 24-well cell culture dish, and analysis was performed after treating cells which had reached 70 % confluence. The relative GFP fluorescence was compared qualitatively, but GFP fluorescence was not used to gate the cells as the primary comparison is the presence or absence of a population shift.

Gating strategy

FSC/SSC was used gate out debris and to identify singlet HEK293 cells and compare GFP fluorescence by histogram.

☒ Tick this box to confirm that a figure exemplifying the gating strategy is provided in the Supplementary Information.
